# Assessing and correcting for regression toward the mean in deviance-induced social conformity

**DOI:** 10.3389/fpsyg.2015.00669

**Published:** 2015-05-22

**Authors:** Robert Schnuerch, Martin Schnuerch, Henning Gibbons

**Affiliations:** ^1^Department of Psychology, University of BonnBonn, Germany; ^2^Department of Psychology, University of MannheimMannheim, Germany

**Keywords:** social influence, conformity, deviance, regression toward the mean, social neuroscience

## Abstract

Our understanding of the mechanisms underlying social conformity has recently advanced due to the employment of neuroscience methodology and novel experimental approaches. Most prominently, several studies have demonstrated the role of neural reinforcement-learning processes in conformal adjustments using a specifically designed and frequently replicated paradigm. Only very recently, the validity of the critical behavioral effect in this very paradigm was seriously questioned, as it invites the unwanted contribution of regression toward the mean. Using a straightforward control-group design, we corroborate this recent finding and demonstrate the involvement of statistical distortions. Additionally, however, we provide conclusive evidence that the paradigm nevertheless captures behavioral effects that can only be attributed to social influence. Finally, we present a mathematical approach that allows to isolate and quantify the paradigm’s true conformity effect both at the group level and for each individual participant. These data as well as relevant theoretical considerations suggest that the groundbreaking findings regarding the brain mechanisms of social conformity that were obtained with this recently criticized paradigm were indeed valid. Moreover, we support earlier suggestions that distorted behavioral effects can be rectified by means of appropriate correction procedures.

## Introduction

Social influence clearly is one of the classic issues of social psychological research (Asch, 1955; Crutchfield, 1955). Adding to a vast body of behavioral studies on its mechanisms and nature (Cialdini and Goldstein, 2004), scholars have recently begun to investigate it using neuroscientific methodology (Falk et al., 2012; Izuma, 2013; Cascio et al., 2015). In particular, various studies have broadened the perspective on conformity (i.e., aligning one’s judgments to those of others), providing insight that is valuable both for the brain sciences and for social psychology (Schnuerch and Gibbons, 2014). To study the neurophysiological principles of conformity, several studies have implemented innovative designs that allow to investigate the perception of one’s deviance from descriptive social norms and the influence of this deviance on subsequent individual judgment (Klucharev et al., 2009, 2011; Zaki et al., 2011; Kim et al., 2012; Shestakova et al., 2013; Huang et al., 2014; Nook and Zaki, 2015). Using a specifically developed paradigm (Klucharev et al., 2009), it was shown, and repeatedly replicated, that majority influence involves the general neural principles of reinforcement learning (Falk et al., 2012): detecting one’s agreement with the majority leads to patterns of activity typically observed in reward processing, while perceiving one’s deviance from the group entails neural responses associated with error and punishment processing (Klucharev et al., 2009, 2011; Shestakova et al., 2013; Stallen et al., 2013).

Only very recently, the experimental approach that was used in most of the studies on the reinforcement-learning principles of social conformity has been called into question as it evidently invites the contribution of regression toward the mean (RTM; Yu and Chen, 2015). In the typical paradigm (Klucharev et al., 2009), participants rated the attractiveness of a series of faces on a Likert scale and saw, after each of their own ratings, how an ostensible group of others had previously rated the respective face. Thus, participants learned about the possible discrepancy (or agreement) between their own and the group’s ratings. In a surprise retest session, 30 min after the initial session, participants were later asked to rate all faces again, this time without any feedback about group judgment. Participants’ second ratings severely decreased for images that the group had previously rated less favorably than they themselves, and ratings greatly increased when group judgment had been above their own, which is taken as evidence of conformity to descriptive group norms.

However, as discussed in detail only recently by Yu and Chen (2015), the experimentally manipulated ostensible group judgment can only deviate upward (i.e., be higher than the individual’s) when participants’ initial ratings are sufficiently low. Also, group judgment can only deviate downward (i.e., be lower than the individual’s) when initial ratings are high. Thus, the independent variable that is assumed to elicit the behavioral effect is severely constrained. More specifically, group deviation from individual judgment is *confounded* with the level of the initial rating. Unfortunately, the level of an initial rating has its own substantial effect on the repetition of this rating, as measurements above or below the mean tend to regress in the opposite direction to approach the mean (Tversky and Kahneman, 1974; Cutter, 1976; Blomqvist, 1987; Stigler, 1997; Pezdek and Eddy, 2001). Thus, what is interpreted as an effect of the individual’s deviance from the group might actually reflect the fact that initially extreme ratings were simply less extreme during a second assessment: high ratings, typically followed by lower group judgment, decrease toward the mean by default (and this direction just so happens to be the direction of the group’s deviating judgment). Likewise, low initial ratings, most frequently followed by higher group judgments, naturally regress to be higher in a second assessment (Yu and Chen, 2015).

Using an elegant experimental approach, Yu and Chen (2015) demonstrated RTM in the deviance-based conformity paradigm (Klucharev et al., 2009). Yu and Chen (2015) showed that the same effect typically observed in this paradigm occurred even in the complete absence of the seemingly critical manipulation. After each of participants’ judgments, they determined the group’s judgment, yet did not show it to the participant, who merely rated all faces and was later asked to rate them all again. Crucially, conformal adjustment as a function of participants’ “previous deviance” was found, even though this deviance was completely unbeknownst to the participants. This effect vanished when the level of participants’ initial ratings was controlled for. According to Yu and Chen (2015), this clearly demonstrates that RTM caused the effect that would have been attributed to the social-feedback manipulation under normal conditions.

This recent study by Yu and Chen (2015) exemplifies the need for careful methodological considerations and, more specifically, the necessity to control for RTM. As acknowledged by the authors, though, their study does not clarify whether the deviance-based conformity paradigm indeed captures true conformity effects. Even though the sham-manipulation effect indicates that the paradigm evokes a notable RTM-induced effect (Yu and Chen, 2015), it is still conceivable that RTM *added* to the actual social-influence effect rather than accounting for the entire behavioral effect. There is indeed evidence indicating that this might be the case. Regression effects were controlled for using *post hoc* procedures in previous studies (such as removing trials from the analysis or adding initial ratings as covariates in the analysis), and an effect of deviance from the group on subsequent rating changes still emerged (Zaki et al., 2011; Huang et al., 2014; Nook and Zaki, 2015). In all of these studies, the authors controlled for the level of the initial rating, as similarly proposed by Yu and Chen (2015).

One might argue that roughly controlling for the level of the initial rating is not the ultimate way to investigate possible remainders of the social-influence effect in the deviance-based paradigm. In fact, one should consider the possibility that leveling initial judgments or removing extreme ones distorts the measurement of conformity if conformity itself is not independent of the level of one’s initial rating. For example, a special property of the deviance-based paradigm might be that participants attend vigilantly to the following group judgment whenever they have just given a rather *extreme* initial rating. In such situations, social proof would seem particularly desirable to the person. Increased attention to the group judgment on a particular trial, however, will likely entail a greater conformal adjustment. A conformity estimate that is based solely or mainly on *moderate* initial ratings might therefore lead to an underestimation of the social-conformity effect. Whatever the actual mechanisms, if not only RTM, but also conformity is systematically related to the level of the initial ratings, any *post hoc* correction procedure that completely purges the influence of the initial rating would be inaccurate. Consequently, alternative strategies for correction should be tested.

There is general consensus that the best approach to controlling for RTM when taking measures repeatedly is to include a control group (Barnett, 2004; Yu and Chen, 2015). By assessing whether the effect in an experimental group, in which the regular manipulation is used, exceeds the effect in a control group, in which a sham manipulation is used that leads to the same degree of RTM, yet does not contain the crucial social-influence manipulation (Yu and Chen, 2015), one can assess indubitably whether there is a true conformity effect. In the present study, we therefore implemented a straightforward and highly expedient control-group design for the first time in this line of research.

In addition to clarifying whether there is a conformity effect in the deviance-based paradigm *at the group level*, we would like to introduce a practical approach that might allow us to quantify and remove this effect at the level *of the individual participant*. More precisely, we propose a novel strategy by which the control group can be used to correct for RTM in the experimental group. Based on the results from the control group, one can carefully assess how rating changes carried by natural RTM can be predicted on the basis of initial ratings. It is reasonable to assume that initial ratings are related to subsequent RTM (Zaki et al., 2011; Huang et al., 2014; Yu and Chen, 2015). We posit that the exact influence of initial ratings on subsequent RTM should be quantified in a control-group design. Applying hierarchical linear modeling to the data of the control group, one can determine an equation and parameters that allow to predict for each follow-up rating the change in rating that is expected due to RTM alone. Subsequently, one can apply this model to the data in the experimental group to estimate an RTM-corrected rating change for each item in each participant.

It should be noted that such a minute correction procedure is useful for several reasons. First, it allows to subsequently assess a corrected conformity score for each participant. In neuroscience research, it is often of vital interest to correlate physiological and behavioral effects across participants (Klucharev et al., 2009; Shestakova et al., 2013; Schnuerch et al., 2014; Nook and Zaki, 2015). Likewise, studies on the role of genetics in social influence essentially depend on valid measures of individual conformal adjustment (see Falk et al., 2012). Second, conformity research has typically focused on investigating moderators of socially influenced behavior, such as the nature of the object that is being judged (see, e.g., Spears et al., 2009) or its ambiguity (see, e.g., Germar et al., 2014). As long as the observed effects in the deviance-based paradigm are artificially inflated by a distortion that should be similar across different conditions, it is particularly difficult to uncover or quantify any such differences, even if they do exist. Therefore, we believe that there is ample reason to explore and discuss possible procedures that allow not only to pinpoint, but also to quantify and correct for the unwanted contribution of RTM (Yu and Chen, 2015). Consequently, a corrected, and thus more precise, estimate of each participant’s behavioral tendency to conform is pivotal. The approach proposed in the present paper allows to derive such an approximation to a carefully corrected conformity score in the deviance-based paradigm.

## Materials and Methods

### Participants

Fifty-four female undergraduates from the University of Bonn (mean age: 22 years) participated in exchange for course credit. Prior to participation, they gave written informed consent. As in previous studies, only females were run to focus on within-gender, rather than more mating-related, attractiveness judgments (Klucharev et al., 2009, 2011; Shestakova et al., 2013). All procedures were approved by the local ethics committee.

### Stimuli and Apparatus

A total of 180 photographs of female faces were presented over the course of the experiment. The images were taken from the same collection that was previously used in very similar investigations (Klucharev et al., 2009, 2011; Zaki et al., 2011; Shestakova et al., 2013). All faces were presented individually with a six-point rating scale placed below the image. The experiment was programmed and run on Presentation software (Neurobehavioral Systems, Berkeley, CA, USA). All data were processed and analyzed on R (R Core Team, 2014).

### Procedure

We adapted the paradigm developed by Klucharev et al. (2009) and combined it with the approach of Yu and Chen (2015). While one group of randomly selected participants performed the regular task (Experimental Group), the other half did not see any group feedback and merely rated all images twice, in two separate sessions (Control Group). All participants were informed that they took part in a study on the processing of facial beauty and were asked to rate a series of portraits of females. Participants in the Experimental Group were additionally told that they would see “group feedback” directly after each of their own ratings, namely the rounded average assessment of the current face as given by a group of previous participants. For each participant, the experiment consisted of two major parts (first and second rating session), each of which comprised 180 trials.

**FIGURE 1:**
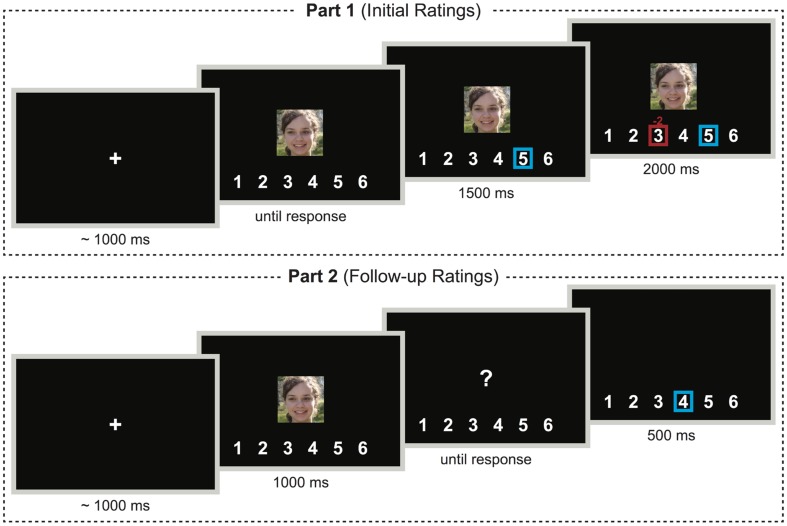
**Schematic illustration of the structure of trials in the first and second part of the experiment in the Experimental Group**. In the Control Group, everything was the same, except that, during the first part, the red frame and number indicating the group’s judgment and its deviation from individual judgment were never shown. Note that this is for demonstration purposes only; different portraits were shown and details are not drawn to scale. Photographs courtesy of David Niblack (www.imagebase.net).

As depicted in **Figure 1**, in the first part (initial ratings), participants judged the attractiveness of the female faces on a Likert scale ranging from 1 (*not attractive at all*) to 6 (*very attractive*). Participants saw each face and entered their rating by keypress. The response was visualized by a blue square surrounding the corresponding number on the scale depicted below the face. In the Control Group, this display remained on-screen for the next 3500 ms. In the Experimental Group, however, a red square, marking the response of an ostensible group of previous participants, appeared around another or the same number on the scale after 1500 ms. Above the square, a small number indicated the degree of deviation between individual and group judgment. The to-be-evaluated face was constantly shown above the scale and the squares. The whole display was shown for 2000 ms, after which the next trial commenced with a fixation cross. Thus, the same time elapsed between participants’ ratings and the next trial in both groups. Also, the display was almost identical, except for the additional square and the small number indicating the group’s response and deviation in the Experimental Group.

Deviation was experimentally manipulated to be -2, -1, +1, or +2 (30 times each) or 0 (60 times), in randomized order. When the summation of the initial individual rating and the currently drawn group deviation resulted in a number smaller than one or larger than six, the drawn deviation ^∗^ (-1) was presented instead. For example, when a participant rated a face as a 5 and +2 was drawn as group deviation, group judgment seven was not shown. Instead, a downward deviation of the same size (-2) was chosen, such that three would be presented. The same approach was employed in previous studies (Kim et al., 2012; Schnuerch et al., 2014). To test the mock effect of (invisible) feedback in the Control Group (Yu and Chen, 2015), deviation of the group was determined in each trial in this group just as in the Experimental Group. It was, however, suppressed, such that no actual social manipulation was presented.

In the previously unannounced second part (follow-up ratings), participants rated all 180 faces for a second time in a newly randomized order. Each face was presented for 1000 ms, until it was replaced by an interrogation point prompting participants to enter their rating, which was visualized for 500 ms by a blue square surrounding the respective number. No group feedback was presented in this part.

### Data Analysis

Prior to all analyses, we mean-centered all ratings separately for each individual and each rating session (first and second rating) to correct for typical displacements across separate sessions (Sharot et al., 2012; Huang et al., 2014; Schnuerch et al., 2014; Yu and Chen, 2015). That is, for a given participant, the mean of all of this participant’s ratings in a given session (first or second) was subtracted from each of this participant’s ratings in this session. Subsequently, we assessed rating changes as the difference between the second and first mean-centered rating of each image. The resulting set of 180 rating changes per participant (based on one initial and one follow-up assessment for a total of 180 images) was investigated. As in previous investigations (Klucharev et al., 2009; Zaki et al., 2011; Shestakova et al., 2013; Nook and Zaki, 2015), large and medium deviations were collapsed, reducing the five-level factor to a factor Deviation with levels *peers lower* (deviations -2 and -1), *peers equal* (deviation 0), and *peers higher* (deviations +1 and +2). Individual rating changes were submitted to an analysis of variance (ANOVA) with repeated-measures factor Deviation (peers lower, peers equal, peers higher) and between-subjects factor Group (experimental vs. control).

For all ANOVAs, we report generalized eta-squared (η^2^_G_) as a measure of effect size (Olejnik and Algina, 2003; Bakeman, 2005). Whenever a test of sphericity indicated that the variances of differences between conditions were not homogeneous (Mauchly, 1940), degrees of freedom were corrected by means of the procedure proposed by Greenhouse and Geisser (1959), and uncorrected values and the correction factor ε are reported. For *t*-tests, Cohen’s *d* is reported as effect size.

The proposed correction procedure is based on a hierarchical linear model (see Rationale and Derivation of the Correction Formula), which was analyzed using R packages lme4 and lmerTest (Bates et al., 2014; Kuznetsova et al., 2014). Degrees of freedom were based on the Satterthwaite approximation (Satterthwaite, 1946).

## Results

### Effect of Deviation on Rating Changes

In a preliminary analysis, we confirmed the successful randomization of participants’ assignment to the groups (Experimental vs. Control) by comparing their raw (i.e., untransformed) initial ratings. As expected, initial ratings did not differ significantly between groups [Control: *M* = 3.087, *SD* = 0.403; Experimental: *M* = 3.198, *SD* = 0.422; *t*(52) = 0.992, *p* = 0.326, *d* = 0.269].

**FIGURE 2:**
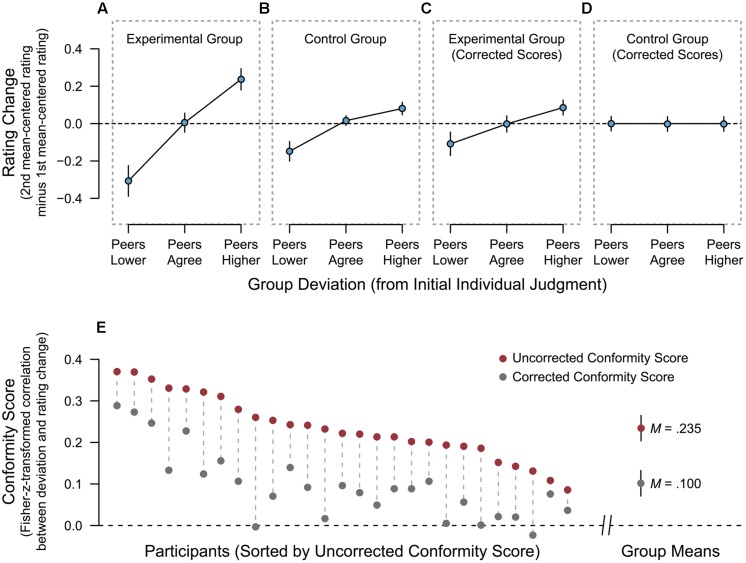
**Effects of the social-deviance manipulation on individual judgment at the group level and for each participant. (A)** Rating changes as a function of the deviation of group judgment from participants’ initial judgment in the Experimental Group, that is, when group judgment is actually presented. **(B)** Rating changes as a function of preceding group deviation in the Control Group, that is, when no group judgment is ever shown. **(C)** Corrected rating changes as a function of preceding group deviation in the Experimental Group. The correction was performed using the formula presented in Section “Rationale and Derivation of the Correction Formula.” **(D)** Corrected rating changes as a function of preceding group deviation in the Control Group. The correction was performed using the formula presented in Section “Rationale and Derivation of the Correction Formula.” **(E)** Individual conformal tendencies for all participants in the Experimental Group, shown as uncorrected and corrected conformity scores. Conformity scores are Fisher-z-transformed within-subject correlations between the group’s deviation and (corrected or uncorrected) rating changes (see Application of the Correction Formula for details). Error bars in all panels **(A–E)** depict the 95% confidence interval of the mean.

In both groups, rating changes depended on the [previously presented (Experimental Group) or drawn, but not presented (Control Group)] deviation of group judgment from individual judgment, as shown by a main effect of Deviation on rating changes [*F*(2,104) = 80.045, *p* < 0.001, η^2^_G_ = 0.590, ε = 0.794]. This effect was further modulated by the group, as indicated by the significant interaction Deviation × Group [*F*(2,104) = 13.077, *p* < 0.001, η^2^_G_ = 0.190, ε = 0.794]. To assess the size of the group-level effect for each group, we performed separate follow-up ANOVAs. As expected, the effect of Deviation on rating changes was larger in the Experimental Group [*F*(2,52) = 52.777, *p* < 0.001, η^2^_G_ = 0.658, ε = 0.768] than in the Control Group [*F*(2,52) = 28.562, *p* < 0.001, η^2^_G_ = 0.497, ε = 0.822]. As the two groups differed only in regard to the social-deviance manipulation, while most likely containing the same degree of RTM, there must have been an effect of this manipulation that exceeded the mere RTM effect in the Experimental Group. **Figures 2A,B** display descriptives for the two groups.

### Correcting Individual Rating Changes

#### Rationale and Derivation of the Correction Formula

One key advantage of the control-group design is the possibility of assessing an item-level estimate for RTM in the given paradigm. As will be demonstrated in the following, this allows to assess a *corrected conformity score* for each participant. Previous research has provided empirical support for the theoretical assumption that, as per RTM, initial ratings should strongly affect follow-up ratings, and thus rating changes (Zaki et al., 2011; Huang et al., 2014; Nook and Zaki, 2015; Yu and Chen, 2015). We argue that this fact (i.e., the natural influence of an initial rating regarding a given item on the follow-up rating of this very item) can be used to estimate more precisely, for each participant and each item, the to-be-expected rating change that is carried by RTM alone. [Note that we refer to *items*, rather than *trials*: although each participant performed a total of 360 trials, exactly 180 independent items (images) were rated twice, such that the initial rating, the follow-up rating, and the rating change are registered and available for analysis for each of these 180 items].

The general approach of our correction procedure is based upon the idea that the Control Group, in which no social-influence manipulation was presented, is ideal to assess the isolated RTM effect of initial ratings on subsequent rating changes. From the Control Group one can derive a hierarchical linear model that allows to predict rating changes on the basis of initial ratings. This model can then be applied to the Experimental Group in order to estimate the expected rating change caused by the level of the initial rating (i.e., RTM). Finally, one can subtract this estimate of the RTM-based rating change from each rating change in order to arrive at a corrected rating-change estimate per item that captures only the influence of group deviation.

To give a brief overview beforehand, we will present two equations that describe how RTM-predicting parameters can be estimated based on the behavior observed in the Control Group (with participants always indexed by *k*). The third equation describes how the previously estimated parameters can be applied to the data observed in the Experimental Group (with participants always indexed by *x*) to predict behavior that should be driven by social influence without the contribution of RTM.

To account for the hierarchical data structure (all 180 items are nested within each participant), we implemented a random-coefficient model (Bryk and Raudenbush, 1992). By use of a hierarchical linear model such as this, we can analyze the data within each participant (Level 1) as well as differences between participants (Level 2). Therefore, the rating change for each item *i* of each Control-Group participant *k* is entered as the dependent variable of the Level-1 model (item level), which can be formulated as

(1) $$c_{ik}=\beta_{0k}+\beta_{1k}r_{ik}+\varepsilon_{ik}$$

where *c_ik_* is the observed rating change, *r_ik_* is the mean-centered initial rating for item *i* of Control-Group participant *k*, β_0_*_k_* represents the individual intercept of participant *k*, β_1_*_k_* denotes the unstandardized effect of the first rating on rating change for participant *k*, and ε*_ik_* represents the normally distributed residual term for item *i* of participant *k*, with ε*_ik_* ∼*N*(0, σ^2^_ε_). In a hierarchical linear model, regression coefficients are allowed to vary between participants (random coefficients), so that each participant practically has their own regression equation. These individual differences are further addressed in Level-2 models. Since the independent variable *r_ik_* is mean-centered within participants, the individual intercept β_0_*_k_* denotes the mean of the dependent variable *c_ik_* for each participant (Bryk and Raudenbush, 1992). As *c_ik_* is the difference between two mean-centered variables (i.e., follow-up and initial rating), it follows that β_0_*_k_* is zero for all participants. Thus, our model is in fact a random-slope, fixed-intercept model with β_0_*_k_* = β_0_ = 0.

The Level-2 model (participant level) describes the random slope of the Level-1 model as a function of an average effect and a normally distributed random error:

(2) $$\beta_{1k}=\gamma_{10}+\delta_{1k}$$

where β_1_*_k_* is the slope of participant *k*, γ_10_ is the average slope across participants, and δ_1_*_k_* is the individual deviation from that mean associated with participant *k*, with δ*_1k_* ∼*N*(0, σ^2^_δ_). Model analysis based on the hierarchical linear model as specified above includes the estimation of fixed coefficients on both levels (i.e., β_0_ and γ_10_) as well as residual variances on both levels (i.e., σ^2^_ε_ and σ^2^_δ_).

As expected, the fixed effect of initial rating, that is, the average slope across participants, was a strong and significant predictor of rating changes in the Control Group [γ_10_ = -0.374, *SE* = 0.019, *F*(1,26.283) = 377.830, *p* < 0.001]. The random-effect analysis of initial rating revealed that the observed slope differed only slightly between participants (σ^2^_δ_ = 0.007). RTM-induced rating changes per item for each participant in the Experimental Group can now be estimated by weighting the initial rating with the extracted average slope γ_10_. As the total (i.e., observed) rating change for each trial is assumed to be the sum of the RTM-induced rating change and the social-influence-induced rating change, the part of the rating change that is due to the social-influence manipulation can be approximated for each item *i* in each Experimental-Group participant *x* as follows:

(3) s^ix=cix−γ10rix⁢

where *ŝ_ix_* is the predicted social-influence-induced rating change (i.e., rating change adjusted for RTM), *c_ix_* is the observed rating change, γ_10_ is the previously determined regression coefficient (i.e., the average slope) for the mean-centered initial rating (see Equation 2), and *r_ix_* is the mean-centered initial rating.

#### Application of the Correction Formula

The above-mentioned model can now be applied to the data in the Experimental Group to correct all rating changes at the level of individual items. Subsequently, items can be aggregated for each deviation condition in each participant, and group-level analyses can be performed in the same way as it is usually done with the *uncorrected* values to assess the overall effect of the manipulation. Such an analysis yields an approximation to the group-level social-influence effect without the contribution of RTM. For the Experimental Group in the present study, the repeated-measures ANOVA with dependent variable corrected rating change (as described for the uncorrected values in Section “Effect of Deviation on Rating Changes”) revealed a significant effect of the 3-level factor Deviation on corrected rating changes [*F*(2,52) = 11.114, *p* < 0.001, η^2^_G_ = 0.297, ε = 0.792]. Holm-corrected pairwise comparisons (Holm, 1979) revealed that rating changes were lower (i.e., more negative) in the peers-lower as compared to the peers-agree condition (*p* = 0.037) and compared to the peers-higher condition (*p* = 0.001). Also, rating changes were higher in the peers-higher than in the peers-agree condition (*p* = 0.008). Although based on a more minute correction procedure, this confirms previous findings indicating that a significant conformity effect emerges in this paradigm, even when the unwanted contribution of RTM is controlled for (Zaki et al., 2011; Huang et al., 2014; Nook and Zaki, 2015; Yu and Chen, 2015). Descriptives at the group level are shown in **Figure 2C**.

Additionally, we scrutinized our own approach by applying it to all rating changes in the Control Group as well. The previously reported effect of the invisible deviation on rating changes in the Control Group (see Effect of Deviation on Rating Changes) can only represent RTM. If the proposed correction algorithm indeed isolates and subducts the contribution of RTM to rating changes, applying it to the Control Group should thus completely eliminate the effect. A repeated-measures ANOVA with dependent variable social-influence-incuded rating change in the Control Group revealed that there was no longer a significant main effect of Deviation, [*F*(2,52) = 0.273, *p* = 0.762, η^2^_G_ = 0.010]. As expected, the correction procedure thus exposes a remaining social-influence effect in the Experimental Group (see **Figure 2C**), while no such effect is observed in the Control Group (see **Figure 2D**).

To estimate *individual* RTM-corrected social-influence effects, we modified an approach that was previously implemented in the deviance-based conformity paradigm (Klucharev et al., 2009; Nook and Zaki, 2015): for each participant separately, we assessed across all items the Pearson’s *r* correlation coefficient for the association between (a) the group’s deviation from the individual [-2, -1, 0, +1, +2] and (b) subsequent rating change. The resulting raw coefficients were transformed to Fisher-*z* scores to guarantee a normal distribution of the values and allow comparison of the estimates (Fisher, 1921; Nook and Zaki, 2015). Crucially, we performed this analysis twice, using two different variables for (b): the unmodified rating changes as they were observed [*c*], and the estimates of the RTM-corrected rating change attributable to social influence [ŝ], computed using the formula depicted in Equation 3. The first correlation provides the uncorrected conformity score for each participant, whereas the second one provides the corrected conformity score. Based on our hierarchical model analysis in the Control Group, γ_10_ was set to -0.374 (see Rationale and Derivation of the Correction Formula).

Uncorrected and corrected conformity scores for all participants are depicted in **Figure 2E**. Note that the degree of correction obviously varies between participants. However, this is one of the strengths of the item-based approach: if a person’s initial ratings scatter widely around their average (e.g., ratings 1 and 6 are used most frequently), then much RTM is highly likely and, thus, a strong correction of subsequent ratings is necessary. If a person’s initial ratings have only a very limited variance (e.g., only ratings 3 and 4 are used), then RTM occurs only rarely and is rather small, such that the correction should be minimal.

Uncorrected scores ranged from 0.085 to 0.370, with a mean of 0.235 (*SD* = 0.078). Across participants, uncorrected conformity scores were significantly larger than zero [*t*(26) = 15.645, *p* < 0.001, *d* = 3.013]. RTM-corrected conformity scores ranged from -0.023 to 0.289, with a mean score of 0.100 (*SD* = 0.084). As expected, uncorrected and corrected scores were strongly correlated [*r*(27) = 0.802, *p* < 0.001], and corrected scores were significantly smaller than uncorrected scores [*t*(26) = 14.166, *p* < 0.001, *d* = 2.726]. However, the corrected scores were still significantly larger than zero [*t*(26) = 5.885, *p* < 0.001, *d* = 1.190], which is consistent with the results from the initial comparison between Experimental and Control Group (see Effect of Deviation on Rating Changes) and the group-level ANOVA on social-influence-induced rating changes (i.e., rating changes corrected according to Equation 3) in the Experimental Group.

## Discussion

In the present study, we investigated the nature of the behavioral effects in the experimental paradigm that is frequently used to study the reinforcement-learning principles of social conformity (Klucharev et al., 2009, 2011; Zaki et al., 2011; Kim et al., 2012; Shestakova et al., 2013; Huang et al., 2014; Nook and Zaki, 2015). Our study is thus a continuation of very recent methodological investigations that demonstrated that the paradigm measures a behavioral effect that is most likely carried by RTM (Yu and Chen, 2015). We aimed to (i) investigate carefully whether the paradigm nevertheless yields a behavioral effect that must be attributed to social influence rather than RTM, and (ii) develop a correction procedure that allows to correct the measure of conformity at the level of the individual participant.

Previously employed *post hoc* corrections (Zaki et al., 2011; Huang et al., 2014), as well as the modulating effects of additional social variables on the compound behavioral effect (Klucharev et al., 2009), indicate that the paradigm indeed captures true conformity effects. To verify and extend these findings, we employed, for the first time in this line of research, the best approach to controlling for RTM (Barnett, 2004; Yu and Chen, 2015): a straightforward control-group design. As expected, we found that the regular paradigm yields a behavioral effect that exceeds the effect that is solely carried by RTM.

The present findings thus invigorate the interpretation of previous work based on the deviance-based paradigm (Klucharev et al., 2009). Admittedly, the brain mechanisms revealed via this approach were mostly independent of the behavioral effects, as they referred to the process of deviance detection (Klucharev et al., 2009, 2011; Kim et al., 2012; Shestakova et al., 2013; Huang et al., 2014) and to the altered representation of value (Zaki et al., 2011). Thus, the unwanted contribution of RTM, which is a phenomenon that pertains to the ratings during the second rating session, rather than to the processing of social deviance or an item’s value, might be seen as somewhat detached from the neural findings. However, from a general theoretical and scientific point of view, it has been strongly advised that neuroscience research base its conclusion about the physiological mechanisms underlying psychological processes upon firm and valid behavioral effects (Amodio, 2010). According to this view, it is quintessential to the interpretation of any neuroscientific findings based on this paradigm that the experimental setup indeed evokes the assumed psychological effects of social influence that register at the behavioral level. The present study, in line with previous *post hoc* correction approaches (Zaki et al., 2011; Huang et al., 2014; Nook and Zaki, 2015), strongly supports this assumption by demonstrating that RTM accounts merely for part of the behavioral effect. The paradigm measures not only unwanted statistical distortions, but also a substantial and relevant aspect of human social behavior. Thus, the novel and intriguing insight into the neural principles of social influence obtained in this paradigm (Klucharev et al., 2009; Zaki et al., 2011; Kim et al., 2012; Shestakova et al., 2013) was based upon a task that indeed induces social influence and captures conformal adjustments. This is particularly noteworthy as these neurophysiological findings have received much attention and are seen as highly relevant advances in social-influence research as well as social and decision neuroscience in general (Rilling and Sanfey, 2011; Falk et al., 2012; Schnuerch and Gibbons, 2014; Cascio et al., 2015). Clearly, they have broadened our perspective on social influence and the mechanisms of the social brain (Klucharev et al., 2009; Kim et al., 2012). The present study substantiates these claims and advances previous methodological caveats (Yu and Chen, 2015) by demonstrating that these groundbreaking findings derived from an experimental setting that clearly instigates the targeted psychological processes.

Importantly, the present work does not only affect the interpretation of previous results, but it also adds to the list of potential correction procedures that were recently proposed (Yu and Chen, 2015) and thus contributes to the improvement of any further implementation of this paradigm. We have presented a feasible mathematical approach by which the size of the true conformity effect can be determined *for each participant individually*. While still allowing an enhanced estimation of the group-level effect, this mainly opens the possibility of relating the effect to external variables across participants. In fact, several scholars using this paradigm did not find correlations between the behavioral effect and neural effects across or within participants (Kim et al., 2012; Shestakova et al., 2013; Huang et al., 2014). We speculate that this null effect might in part be due to the fact that the behavioral measure was an overestimation partly carried by RTM. A cleaner measure of conformity, as proposed in the present study, might turn out to allow finding such relations. This could be particularly relevant for further investigations into the reinforcement-learning principles of conformity, which often involve an individual-differences perspective (Falk et al., 2012). Relating a person’s genetic disposition or their brain activity to behavioral measures of conformity is useful in uncovering the biological foundations of this ubiquitous behavior (Falk et al., 2012, 2014). If the innovative and clearly beneficial deviance-based paradigm is implemented as part of this endeavor, carefully corrected behavioral measures at the level of the individual participant are mandatory. The present study proposes a novel strategy how to do this.

The rationale of the proposed correction approach is straightforward, as it builds upon the ideas of a compound effect that can be rectified and the possibility of deriving a means to predict RTM-induced behavioral changes in this paradigm. Importantly, the procedure targets the lowest level of information in this paradigm, which is the initial and follow-up ratings for each given item. Using the control group as a separate sample to derive an estimate that allows to predict RTM-induced rating changes for each to-be-rated item, one can subtract the influence of RTM from all rating changes in the experimental group. The remaining rating changes are a valid approximation to the actual modulation of individual judgment due to social influence. Crucially, all information that is necessary to generate a prediction model and apply this for correction is at hand: only initial and follow-up ratings in a control group and an experimental group are needed to perform this correction.

Finally, it should be noted that the correction procedure proposed in the present paper would benefit from further testing. Additional experimental investigations, as well as simulation studies, could provide more certainty as to the accuracy of the approach. Although its general notion is rather straightforward, the procedure builds upon assumptions that could be scrutinized to test its validity and stability. For example, we assume that RTM is independent of the critical manipulation (i.e., deviation in the present study), such that any effect observed in the control group is a valid proxy for the expected RTM effect in the experimental group. While this seems plausible (see Barnett, 2004), empirical evidence supporting this assumption would increase the confidence in the procedure’s potency. The basic conditions (e.g., sample sizes, response variability, and scale use) could be manipulated in order to test whether the procedure introduced and tested in this paper is robust to such variations and remains useful under different premises.

## Conclusion

The present study represents a relevant addition to the recently voiced concerns about the deviance-based conformity paradigm (Yu and Chen, 2015). However, beyond clarifying and replicating the unwanted statistical distortion, we have demonstrated its limit. The contribution of RTM to the total behavioral effect is bounded, as revealed by the fact that a substantial social-influence effect remained even if RTM was carefully purged. Moreover, we propose a strategy to estimate conformal tendencies for each pair of judgments of each participant. We therefore conclude that the paradigm can indeed be used, as it allows to harvest the advantage of analyzing behavioral and neural consequences of detecting agreement and disagreement with one’s judgments. In fact, the paradigm continues to be implemented in the social-neuroscience community (Huang et al., 2014; Nook and Zaki, 2015), which emphasizes the need for a clear understanding of the paradigm and its pitfalls. However, it is equally important to recognize the limitations of these problems and develop appropriate strategies to correct for them.

## Conflict of Interest Statement

The authors declare that the research was conducted in the absence of any commercial or financial relationships that could be construed as a potential conflict of interest.
